# Dietary patterns and associated lifestyles in individuals with and without familial history of obesity: a cross-sectional study

**DOI:** 10.1186/1479-5868-3-38

**Published:** 2006-10-31

**Authors:** Ann-Marie Paradis, Louis Pérusse, Marie-Claude Vohl

**Affiliations:** 1 Department of Food Science and Nutrition, Laval University, Quebec, Canada, and Lipid Research Center, CHUQ-CHUL Pavilion, 2705 Laurier Blvd, TR-93, Sainte-Foy, Quebec, G1V 4G2, Canada; 2 Department of Social and Preventive Medicine, Laval University, Quebec, G1K 7P4, Canada

## Abstract

**Background:**

Familial history of obesity (FHO) and certain dietary habits are risk factors for obesity. The objectives of this cross-sectional study were 1) to derive dietary patterns using factor analysis in a population of men and women with and without FHO; 2) to compare mean factor scores for each dietary pattern between individuals with and without FHO; and 3) to examine the association between these patterns and anthropometric, lifestyle and sociodemographic variables.

**Methods:**

A total of 197 women and 129 men with a body mass index <30 kg/m^2 ^were recruited. A positive FHO (FHO+) was defined as having at least one obese first-degree relative and a negative FHO (FHO-) as no obese first-degree relative. Dietary data were collected from a food frequency questionnaire. Factor analysis was performed to derive dietary patterns. Mean factor scores were compared using general linear model among men and women according to FHO. Regression analyses were performed to study the relationship between anthropometric, lifestyle and sociodemographic variables, and each dietary pattern.

**Results:**

Two dietary patterns were identified in both men and women : the Western pattern characterized by a higher consumption of red meats, poultry, processed meats, refined grains as well as desserts, and the Prudent pattern characterized by greater intakes of vegetables, fruits, non-hydrogenated fat, and fish and seafood. Similar Western and Prudent factor scores were observed in individual with and without FHO. In men with FHO+, the Western pattern is negatively associated with age and positively associated with physical activity, smoking, and personal income. In women with FHO-, the Prudent pattern is negatively associated with BMI and smoking and these pattern is positively associated with age and physical activity.

**Conclusion:**

Two dietary patterns have been identified among men and women with and without FHO. Although that FHO does not seem to influence the adherence to dietary patterns, results of this study suggest that anthropometric, lifestyle and sociodemographic variables associated with dietary patterns differ according to FHO and gender.

## Background

Familial history of obesity (FHO) increases the risk of offspring being obese [1-6]. Moreover, it is suggested that dietary habits might be also involved in the development of obesity. Indeed, the role of individual dietary components has been the focus of considerable research in the field of obesity [7-9]. However, since foods are consumed in combinations, several authors have proposed to analyze food consumption as dietary patterns [10-13]. A factor analysis approach used to derive dietary patterns reduces the complexity of diets to a few important foods and has the ability to integrate complex and subtle interactive effects of many dietary exposures [13-15]. Moreover, by using this method it is possible to examine distinct dietary patterns reflecting different dietary habits which may be related to the development of obesity.

Only few studies examined dietary patterns and obesity. However, Newby et al. [16] has examined prospectively whether eating patterns are related to anthropometric changes in men and women in the Baltimore Longitudinal Study of Aging. This study showed that a dietary pattern rich in reduced-fat dairy products and high-fiber foods is inversely associated with annual change in body mass index (BMI) in women and inversely associated with annual difference in waist circumference in both women and men. Knowing that certain dietary patterns may lead to a smaller weight gain [16] and that individuals with FHO are at high risk of obesity [1-6], it appears imperative to investigate the relationship between FHO and dietary patterns. The objectives of this cross-sectional study were 1) to derive dietary patterns using factor analysis in a population of men and women with and without FHO; 2) to compare mean factor scores for each dietary pattern between individuals with and without FHO; and 3) to examine the association between these patterns and anthropometric, lifestyle and sociodemographic variables.

## Methods

### Study population and study design

Participants of the present study were adults aged between 18 to 55 years and a BMI <30 kg/m^2^. We chose to investigate a population with a BMI<30 kg/m^2 ^because we wanted to know whether non-obese individuals with FHO counterbalance the genetic effect, and maintain a healthy body weight by adopting healthier nutritional habits than subjects without FHO. Subjects were recruited in the Quebec City metropolitan area through advertisements in local newspapers and radio stations, and by electronic messages sent to university and hospital employees. A trained research assistant conducted a 15-minute telephone interview with people who responded to the advertisement messages. The assistant asked the participant to report their body weight, height, age and FHO. FHO was assessed by asking to the participant whether he/she has obese first-degree biological relatives (father, mother, brother and sister). FHO was considered to be positive if participant had at least one or more obese (BMI≥30 kg/m^2^) first-degree relatives (parents and siblings). FHO was considered negative if no first-degree relatives had a BMI≥30 kg/m^2^. Total number of biological brothers and sisters was also recorded. Following the interview, eligible participants were invited to come at the laboratory to fill some questionnaires about sociodemographic characteristics and lifestyle variables. Moreover, they met a trained research assistant to collect anthropometric measurements and a dietitian to complete a validated food frequency questionnaire (FFQ) [17]. Participants received 25 dollars (Canadian) to cover transportation and parking expenses after appointment. Enrolment of the subjects took place between May 2004 and December 2004. Subjects who had: a BMI≥30 kg/m^2 ^(n = 10), incomplete information about FHO (n = 6), extreme nutritional values (based on mean ± 4 SD) (n = 1), AIDS (n = 1), and those who were pregnant (n = 1) or homeless (n = 1) were excluded. The final study sample consisted of 129 men and 197 women. All subjects gave their written consent to participate into this study which has been approved by the Ethics Committee of Laval University.

### Sociodemographic characteristics and lifestyle variables

A standardized questionnaire was completed by each participant to obtain information about age, personal income and smoking status. This questionnaire was checked immediately to ensure that the participant filled all questions. Moreover, leisure time activity was estimated using the Minnesota Leisure Time Physical Activity Questionnaire [19]. This instrument has been validated, the Minnesota Leisure Time Physical Activity Questionnaire showed moderate correlations with items from a physical activity record (*r *= 0.47) and heavy intensity activities (*r *= 0.52). This questionnaire was administered by a trained interviewer who was provided with detailed instructions and a list of very clearly defined physical activities. Briefly, participants were asked to identify activities performed during the last year. They had to give the average number of times per month they practiced each physical activity and the average duration (minutes) spent at each time they performed this activity. Time spent in each activity was multiplied by the intensity code (corresponding to the kilocalories (kcal) expended per minute) specific to each activity, and then summed over all activities obtaining a value of overall daily kcal/d.

### Anthropometric measurements

Participants were standing and dressed in light indoor clothing without shoes for anthropometric measures. Beam Scale with height rod graduated in centimetres was used (Detecto, Webb City, USA) to obtain a measure of weight and height. Weight was measured to the nearest 0.1 kg and height was measured to the nearest 0.5 cm. The scale was calibrated before the examination. BMI was computed as weight in kilograms divided by height in meters squared. To minimize variations in anthropometric measurements, all measurements were obtained by the same experienced staff member.

### Dietary assessment and food groupings

Dietary intake over the past month was assessed by a 91-item FFQ administered by a dietitian. This FFQ has been previously validated in French Canadian men and women. Briefly, mean value for intake of most nutrients assessed by the FFQ and the 3-day food record were not statistically different. Energy-adjusted correlation coefficients for the principal macronutrients ranged from 0.36 for proteins to 0.60 for carbohydrates [17]. The FFQ was structured to reflect food habits of the Québec population. Participants were asked how often they consumed each item per day, per week, per month or none at all during the last month. Many examples of portion size were provided for a better estimation of the real portion consumed by the subject. The evaluation of the food intake data was performed by grouping together similar food items. The food grouping scheme was based on similarity of nutrient profiles or culinary usage among foods and was similar to the one used in previous studies [10]. Consideration was also given to groups used in other studies to maintain consistency among studies [14,18]. Some individual food items were classified individually if their composition differs substantially from that of other foods (for example, pizza or eggs) or they were assumed to represent distinct dietary habits (e.g. liquor, wine, beer and French fries). Thirty-seven separate food groups were used in analyses to identify dietary patterns (Table 1).

**Table 1:** Food groupings used in the dietary pattern analysis^a^.

Food groups	Food in the group
Processed meats	Bacon, sausage, hot dogs, salami, bologna, ham, pepperoni, liver paste
Red meats	Beef, veal, lamb, pork, game and horse
Organ meats	Liver or other organ meat such as heart, and kidney
Fish and other seafood	Fish and sea food (all kinds)
Poultry	Chicken and turkey with or without skin
Eggs	Eggs including scrambled, fried, omelets, hard-boiled eggs and quiche
Butter	Salted and unsalted butter
Hydrogenated margarine	Hydrogenated margarine (all kinds)
Non-hydrogenated fat	Non-hydrogenated margarine; oil and salad dressing (all kinds)
Reduced- or low- fat dairy products	Skim, 1% or 2%-fat milk and yogurt
High-fat dairy products	Whole milk; cheese (all kinds); cream; ice cream; yogurt (> 2%-fat)
Hard liquor	Hard liquor such as tequila, gin, vodka, scotch, rum, whiskey
Wine	Red or white wine
Beer	Beer or low alcohol beer
Tea	Regular tea with caffeine, decaffeinated or herbal tea
Coffee	Regular coffee with caffeine or decaffeinated
Fruits	All fruit including fresh, frozen, dried, compote (stewed) or canned
Fruit juices	All fruit juices without additionned sugar
Vegetables	All vegetables
Vegetable juices	Vegetable and tomato juices
Legumes	Lentils, chickpeas, beans, peas soup; soy product including tofu, soy milk
Potatoes other than French fried	Potatoes
French fries	French fries and fried potatoes
Whole grains	Whole-wheat, whole-grain and other multigrain breads, bagels, tortillas, English muffins and pita; wheat or whole-grain pasta and brown rice; oatmeal and cream of wheat; whole-grain cereal^a^
Refined grains	White breads, bagels, pita, tortillas, English muffins, rice, pasta; muffins (home-made); couscous; pancakes; waffles; granola bar; refined cereal^b^
Pizza	Pizza (all kinds)
Snacks	Potato chips, popcorn, crackers
Nuts	All nuts or seed and nuts butter
Regular soft drinks	Regular soft drinks
Diet soft drinks	Diet soft drinks
Mayonnaise	Regular and low-fat mayonnaise
Cream-based soups	Cream soup (all kinds)
Soups, broth, or bouillon	Noncream, broth-based soups
Desserts	Cookies, pies, pudding, cakes, doughnuts, croissants, pastries, muffins (commercial), coated granola bar, frozen treats (ice cream sandwichs, fudge, ice cream bars).
Sweets	Table sugar, brown sugar, maple and corn syrup, honey, jam, candy, fudge, maple taffy, chocolate bar or pieces and candy bar with chocolate
Condiments	Ketchup, mustard, barbecue sauce, soy sauce
Meats pies	Chicken, meat and, salmon pies
Supplement products	Nutribar^® ^and Slim Fast^® ^bars, Thirst quencher

^a^Participants were requested to recall how often, on average, they consumed a given amount of each food during the past month (ranging from [not consumed] to [every day]). Examples of portion size representing 1 standard serving such as 1 slice, 1 tablespoon (15 mL), 1 cup (250 mL), 1 oz of cheese or meat were provided for a better estimation of the real portion consumed by the subject.

^b^Cereal that contain > 2.0 g fiber/serving are in the whole grains

^c^Cereal that contain ≤ 2.0 g fiber/serving are in the refined grains

### Statistical methods

All analyses were performed separately for men and women. General linear model was applied to compare means of each characteristic according to sex and FHO. To identify food patterns in our study population, exploratory factor analysis using the FACTOR procedure were conducted. The factors were orthogonally transformed by using Varimax rotation to achieve structure with independent (nonoverlapping) factor and greater interpretability. Components with an eigenvalue > 1, Scree test and the interpretability of the factors were considered in determining the number of factors to retain. Food groups with positive loadings contribute to the dietary pattern; food groups with negative loadings are inversely associated with the dietary pattern. Food groups with absolute factor loadings ≥0.30 were considered as significantly contributing to the pattern. Dietary pattern scores were calculated by summing the standardized intake of food groups, weighted by the factor loading of the food groups. These scores corresponded to the degree to which each subject conformed to a given dietary pattern. A score was then assigned to each participant for each dietary pattern. In men and women, energy adjusted mean factor scores were compared using general linear model among groups divided on the basis of FHO. Regression analyses were performed separately by gender and FHO to study the relationship between lifestyle variables and sociodemographic and anthropometric characteristics with each dietary patterns. The characteristics used in the analyses were: age, physical activity during leisure time, smoking status, personal income and BMI. The annual personal income was divided into four groups: <12000$, 12 000$–29 999$, 30 000$–49 999$, and >50000$ (expressed in Canadian dollars). The educational level was not included in the model because it had a high degree of collinearity with personal income. All statistical analyses were performed in SAS statistical software, version 8.2 (SAS Institute Inc, Cary, NC) and statistical significance was defined as p < 0.05.

## Results

### Descriptive characteristics

Characteristics of men and women are presented in Table 2. Subjects with FHO+ were older and had a higher BMI (men only) than those with FHO- (*P *< 0.05). Differences in BMI (men only) remained statistically significant after adjustment for age. In both men and women, no statistically significant differences were observed for physical activity, smoking status and personal income. The general characteristic of the diet of the participants were 50% of total energy intake from carbohydrates, 16% from protein and 33% from fat (13.7% of total energy from monounsaturated fatty acids, 11.2% from saturated fatty acids, 5.6% from polyunsaturated fatty acids) (data not shown). No significant difference was observed in macronutrient intakes between individuals with and without FHO.

**Table 2:** Characteristics of men and women among familial history of obesity (FHO)^a^.

	Men		Women	
	
	FHO+ (N = 49)	FHO- (N = 80)	FHO+ (N = 100)	FHO- (N = 97)
Age, y	36.2 ± 10.8	32.4 ± 10.0*	38.0 ± 12.1	32.5 ± 10.3***
Body mass index (BMI), kg/m^2^	25.2 ± 2.0	23.9 ± 2.8*	23.0 ± 2.5	22.3 ± 2.3
Physical activity during leisure time, kcal/day	367 ± 340	373 ± 259	330 ± 259	285 ± 152
Smokers^b^, n(%)	6 (12)	8 (10)	7 (7)	10 (10)
**Personal income ($)**^c^				
<12000, n (%)	14 (29)	29 (37)	27 (28)	36 (37)
12000 – 29999, n (%)	13 (27)	13 (16)	18 (18)	21 (22)
30000 – 49999, n (%)	10 (20)	19 (24)	34 (35)	29 (30)
≥50000, n (%)	12 (24)	18 (23)	19 (19)	11 (11)

^a^General linear model was applied to compare means according to gender and FHO.

^b^Missing value for two men and nine women.

^c^Personal income is expressed as Canadian dollar and was not available for one man and two women.

* Significantly different from the FHO+ group (*P *< 0.05).

*** Significantly different from the FHO+ group (*P *< 0.001).

### Dietary patterns

Factor analysis suggested 2 main dietary patterns from men and women. Factor-loading matrices for these dietary patterns are listed in Table 3. The factor loading of a food increases as the contribution of that food to the corresponding dietary pattern score increases. In men and women, the factor principally characterized by a higher consumption of red meats, poultry, processed meats, refined grains, and dessert was labelled Western pattern and the factor principally characterized by greater intakes of vegetables, fruits, and fish and other seafood was labelled Prudent pattern. Dietary patterns explained 19.9% and 17.2% of the variance in dietary intake in men and women, respectively.

**Table 3:** Factor loading matrix for the two major dietary patterns in healthy men and women.

	Men		Women	
	
Food groups	Factor 1 (Western pattern)	Factor 2 (Prudent pattern)	Factor 1 (Prudent pattern)	Factor 2 (Western pattern)
Red meats	**0.78**^a^	-0.10	0.10	**0.80**
Butter	**0.62**	-0.01	-0.10	0.07
Poultry	**0.59**	0.01	0.09	**0.63**
High-fat dairy products	**0.56**	0.27	0.13	-0.08
Processed meats	**0.55**	-0.21	-0.04	**0.68**
Potatoes other than French fried	**0.47**	-0.18	-0.05	0.15
Refined grains	**0.46**	-0.01	-0.07	**0.38**
Condiments	**0.44**	-0.08	-0.07	0.11
French fries	**0.44**	-0.25	-0.07	0.12
Mayonnaise	**0.36**	-0.09	-0.11	0.09
Desserts	**0.33**	0.08	-0.10	**0.30**
Vegetables	0.02	**0.76**	**0.65**	0.06
Fruits	-0.23	**0.68**	**0.73**	0.14
Non-hydrogenated fat	0.05	**0.62**	0.27	0.19
Fish and other seafood	-0.21	**0.33**	0.33	0.02
Wine	0.06	**0.30**	0.13	-0.03
Nuts	-0.04	0.09	**0.48**	-0.11
Legumes	-0.27	0.26	**0.47**	**-0.41**
Whole grains	-0.11	0.17	**0.41**	-0.09
Organ meats	0.20	0.10	**0.37**	0.24
Fruit juices	0.03	-0.02	**-0.33**	0.05
Beer	0.27	-0.08	-0.11	0.07
Sweets	0.27	0.07	0.02	-0.16
Tea	-0.20	0.23	0.26	-0.02
Eggs	0.18	-0.11	0.02	0.05
Snacks	0.17	0.03	0.08	0.03
Soups, broth, or bouillon	-0.17	0.16	0.09	0.01
Reduced- or low- fat dairy products	0.16	-0.05	0.16	0.11
Regular soft drinks	0.12	-0.19	-0.11	0.13
Meats pies	-0.11	-0.11	0.13	0.04
Diet soft drinks	-0.10	-0.09	-0.11	0.03
Cream-based soups	0.09	-0.07	0.14	0.05
Pizza	0.09	-0.19	-0.04	0.04
Supplement products	0.08	-0.07	0.15	0.03
Coffee	-0.06	0.06	0.16	0.17
Hard liquor	0.02	0.29	0.14	-0.01
Vegetable juices	-0.01	0.01	0.01	0.01
Hydrogenated margarine	0.00	-0.05	-0.01	-0.01
Variance explained (%)	12.7	7.2	9.2	8.0

^a ^Factor loadings ≥0.30 in bold are marked in bold.

### Factor scores

Mean factor scores adjusted for energy intake of the two dietary patterns (Western and Prudent) were compared among men and women according to FHO (data not shown). In men and women, standardised mean factor scores adjusted for energy intake of subject with FHO+ were not statistically different from those with FHO- (*P *> 0.05) (data not shown). However, a large interindividual variability was noted in factor scores for each dietary pattern.

### Factors associated with the adherence to dietary patterns

Regression analyses of anthropometric, lifestyle and sociodemographic variables associated with the adherence to both patterns were conducted in men and women according to FHO. In these analyses, the factor score from each dietary pattern was modeled as a continuous variable. The regression coefficients and confidence intervals (95%) for each variable associated with the adherence to the Western and the Prudent pattern are shown in Table 4 and Table 5, respectively. The Western pattern was negatively associated with age and positively associated with physical activity, smoking and personal income in men with FHO+. The Prudent pattern is positively associated with physical activity in men with FHO+. In women with FHO-, the Prudent pattern is negatively associated with BMI and positively associated with age and physical activity.

**Table 4:** Factors associated with the adherence to a Western dietary pattern^a ^in men and women.

	MEN				WOMEN			
	
	FHO+ (N = 49)		FHO- (N = 80)		FHO+ (N = 100)		FHO- (N-97)	
	
	β	CI (95%)	β	CI (95%)	β	CI (95%)	β	CI (95%)
Age (y)	-0.05	-0.08 to -0.02**	-0.002	-0.036 to +0.031	+0.02	-0.003 to +0.037	+0.02	-0.01 to +0.04
Physical activity during leisure time (kcal/day)	+0.0007	+0.0000 to +0.001*	+0.0008	-0.0002 to +0.0017	-0.0003	-0.001 to +0.0004	-0.0002	-0.002 to +0.001
Current smoker								
No	0 (ref)		0 (ref)		0 (ref)		0 (ref)	
Yes	+0.75	+0.05 to +1.44*	+0.48	-0.40 to +1.36	-0.41	-1.21 to +0.40	+0.29	-0.36 to +0.93
Personal income ($)								
<12000	0 (ref)		0 (ref)		0 (ref)		0 (ref)	
12000 – 29999	+0.01	-0.59 to +0.62	+0.13	-0.65 to +0.91	+0.43	-0.19 to +1.05	-0.34	-0.87 to +0.19
30000 – 49999	+0.52	-0.30 to +1.34	+0.37	-0.32 to +1.06	+0.16	-0.41 to +0.73	-0.17	-0.72 to +0.39
≥50000	+0.98	+0.19 to +1.77*	-0.07	-0.92 to +0.79	+0.28	-0.44 to +1.00	+0.11	-0.73 to +0.96
Body mass index	-0.01	-0.13 to +0.11	+0.02	-0.08 to +0.11	-0.01	-0.10 to +0.08	+0.03	-0.06 to +0.13

^a^The minimum and maximum values for the variable used as outcome (Western pattern) were -2.86 and +2.44 for men and -3.27 and +2.25 for women

β = regression coefficient (a positive coefficient implies a higher adherence to the pattern), CI = confidence interval.

*P < 0.05

**P < 0.01

**Table 5:** Factors associated with the adherence to a Prudent dietary pattern^a ^in men and women.

	MEN				WOMEN			
	
	FHO+ (N = 49)		FHO- (N = 80)		FHO+ (N = 100)		FHO- (N-97)	
	
	β	IC (95%)	β	IC (95%)	β	IC (95%)	β	IC (95%)
Age (y)	+0.04	-0.01 to +0.08	-0.002	-0.03 to +0.03	+0.007	-0.01 to +0.03	+0.03	+0.002 to +0.05*
Physical activity during leisure time (kcal/day)	+0.001	+0.0002 to +0.002*	+0.0007	-0.0001 to +0.0020	+0.0003	-0.0005 to +0.0011	+0.002	+0.001 to +0.004***
Current smoker								
No	0 (ref)		0 (ref)		0 (ref)		0 (ref)	
Yes	-0.65	-1.69 to +0.39	-0.05	-0.84 to 0.74	+0.37	-0.50 to +1.23	-0.09	-0.68 to +0.49
Personal income ($)								
<12000	0 (ref)		0 (ref)		0 (ref)		0 (ref)	
12000 – 29999	+0.44	-0.47 to +1.34	-0.24	-0.94 to +0.45	-0.21	-0.88 to +0.44	+0.10	-0.38 to +0.59
30000 – 49999	+0.24	-1.00 to +1.47	-0.28	-0.90 to 0.34	-0.32	-0.93 to +0.30	+0.03	-0.48 to +0.53
≥50000	+0.12	-1.07 to 1.31	-0.18	-0.95 to 0.58	-0.48	-1.25 to +0.29	-0.13	-0.90 to 0.64
Body mass index	-0.03	-0.21 to +0.16	+0.04	-0.04 to +0.12	-0.02	-0.11 to +0.07	-0.09	-0.18 to -0.006*

^a^The minimum and maximum values for the variable used as outcome (Prudent pattern) were -2.17 and +2.47 for men and -2.73 and +2.71 for women

β = regression coefficient (a positive coefficient implies a higher adherence to the pattern), CI = confidence interval.

*P < 0.05

***P < 0.001

## Discussion

Two major dietary patterns were identified in the analysis of the 129 men and 197 women. The Western pattern principally characterized by a high consumption of red meats, poultry, processed meats, refined grains, and dessert as well as the Prudent pattern characterized by a diet rich in vegetables, fruits, and fish and seafood appear to be consistent with a set of food items that has been reported in similar studies [10,11,20-23]. As reported by previous studies [12,24-26], dietary patterns identified from factor analysis were similar for men and women. However, in this study, the dietary pattern which explains a higher percent of variance in food intake is the Western pattern in men (12.7%) and the Prudent pattern in women (9.2%). The percentage of variance (~20%) explained by the first two dietary patterns identified is similar in magnitude to what has been reported in others studies [22,27-31]. It should be noted that the minimal variance explained is largely dependent on the total number of variables included in the analysis.

In the literature, women with a personal history of obesity were less likely to follow a Western pattern and men with a personal history of obesity were more likely to follow a healthy pattern (Spanish-Mediteranean) [32]. Knowing that personal history of obesity influences the observance to dietary patterns, it was reasonable to believe that FHO could also have influenced it. However, there is no significant difference in dietary factor scores between people with and without FHO. These data must be confirmed in other studies in order to firmly conclude that FHO does not positively influence dietary habits.

To our knowledge, it is the first study which reports anthropometric, lifestyle, and sociodemographic variables associated with adherence to dietary pattern according to FHO. We showed that these variables differ according to gender and FHO. Indeed, in the present study, the Western pattern is negatively associated with age and positively associated with smoking, and physical activity in men with FHO+. Those results are consistent with previous studies which concluded that the Western pattern is negatively associated with age [27,29,32,33] and positively associated with smoking [20,23,27,29]. However, our results are inconsistent with those previously reported which have shown that low physical activity [20,23,27,29] was associated with adherence to the Western pattern. In the present study, men with FHO+ in the highest personal income group were more likely to follow the Western pattern. These results are concordant with the results from the Canadian Community Health Survey: Nutrition (CCHS), based on direct measures of height and weight [34]. Indeed, in this survey, men in higher income households were more likely to be obese than were those in the lower-middle income household [34] suggesting that men having a higher personal income could be more likely to follow a Western pattern and to be obese. In women with FHO-, the Prudent pattern is negatively associated with BMI and positively associated with age and physical activity. Similar results regarding the association between an active lifestyle and healthier diet have been reported [29]. It is interesting to point out that none of the studied factors were associated with Western and Prudent patterns in women with FHO+ and in men with FHO-. However, further research is warranted in exploring the possibility that other lifestyle variables affect the adherence to a dietary pattern in these population subgroups.

There are some limitations to the present study. The first one concerns the FHO data. In this study, subjects had to report the presence or not of obese persons in their first-degree relatives. Although that FHO data represents a potential for misclassification, two studies showed that adult offspring's recall of parental height and weight were in good agreement with measured parental height and weight [35,36]. Despite that method of categorization could potentially obscure real differences between groups, the choice to investigate a non-obese population with and without FHO is a major strength of the present study. Indeed, it is well known that having obese relatives increases one's risk for obesity [1,5,6] and that familial patterns of obesity may be explained by familial similarities in eating patterns and dietary composition [37-40]. A second limitation concerns the food data collection. Measurement errors inherent to the use of a FFQ for dietary assessment include underreporting or overreporting of general food intake, selective underreporting or overreporting of intakes of certain foods, or both. However, this bias was also observed in a great majority of nutritional surveys independently of the method used [41]. Moreover, it has been demonstrated that the FFQ is a reproducible and valid tool to identify dietary patterns by factor analysis [10,42] and we used a FFQ validated in our population [17]. Finally, the use of factor analysis to identify dietary patterns represented a limitation. Indeed, results of factor analysis are affected by subjective but important decisions, including the assignment of food items into food groups, the number of factors to extract, the method of rotation and even the labelling of components [43]. Nevertheless, this method offers an opportunity to summarize and refine large amounts of data to a simple descriptive pattern.

In conclusion, two dietary patterns have been identified among men and women with and without FHO. Although that FHO does not seem to the influence adherence to dietary patterns, results of this study suggest that anthropometric, lifestyle and sociodemographic variables associated with dietary patterns differ according to FHO and gender.

## Competing interests

The author(s) declare that they have no competing interest.

## Authors' contributions

AMP performed the statistical analysis of the data and took the primary role in drafting the manuscript. LP and MCV guided the strategy of the data analysis, assisted with the interpretation of the results, and provided critical review of the manuscript. LP and MCV conceived the study. All authors read and approved the final manuscript.
